# Linseed Dietary Fibers Reduce Apparent Digestibility of Energy and Fat and Weight Gain in Growing Rats

**DOI:** 10.3390/nu5083287

**Published:** 2013-08-19

**Authors:** Mette Kristensen, Knud Erik Bach Knudsen, Henry Jørgensen, David Oomah, Susanne Bügel, Søren Toubro, Inge Tetens, Arne Astrup

**Affiliations:** 1 Department of Nutrition, Exercise and Sports, Faculty of Science, University of Copenhagen, 1958 Frederiksberg C, Denmark; E-Mails: shb@life.ku.dk (S.B.); ast@life.ku.dk (A.A.); 2 Department of Animal Science—Molecular nutrition and cell biology, Aarhus University, 8830 Tjele, Denmark; E-Mails: knuderik.bachknudsen@agrsci.dk (K.E.B.K.); henry.jorgensen@agrsci.dk (H.J.); 3 Pacific Agri-Food Research Centre—Summerland, Agriculture and Agrifood Canada, Summerland, British Columbia V0H 1Z0, Canada; E-Mail: dave.oomah@agr.cg.ca; 4 Reduce-Center, 4000 Roskilde, Denmark; E-Mail: st@reduce.dk; 5 National Food Institute, DTU FOOD, 2860 Søborg, Denmark; E-Mail: intet@food.dtu.dk

**Keywords:** dietary fibers, linseed, fat digestibility, obesity

## Abstract

Dietary fibers (DF) may affect energy balance, an effect often ascribed to the viscous nature of some water soluble DF, which affect luminal viscosity and thus multiple physiological processes. We have tested the hypothesis that viscous linseed DF reduce apparent nutrient digestibility, and limit weight gain, in a randomized feeding trial where 60 male, growing, Wistar rats, with an initial weight of ~200 g, were fed different diets (*n* = 10 per group): low DF control (C), 5% DF from cellulose (5-CEL), CEL + 5% DF from whole (5-WL) or ground linseed (5-GL), CEL + 5% DF from linseed DF extract (5-LDF), and CEL + 10% DF from linseed DF extract (10-LDF). Diets were provided *ad libitum* for 21 days. Feed intake and faecal output were measured during days 17–21. Faecal fat excretion increased with increasing DF content and was highest in the 10-LDF group. Apparent fat digestibility was highest with the C diet (94.9% ± 0.8%) and lowest (74.3% ± 0.6%) with the 10-LDF diet, and decreased in a non-linear manner with increasing DF (*p* < 0.001). Apparent fat digestibility also decreased with increased accessibility of DF (5-WL *vs.* 5-GL) and when the proportion of viscous DF increased (5-GL *vs.* 5-LDF). The 10-LDF resulted in a lower final body weight (258 ± 6.2 g) compared to C (282 ± 5.9 g), 5-CEL (281 ± 5.9 g), and 5-WL (285 ± 5.9 g) (*p* < 0.05). The 10-LDF diet reduced body fat compared to 5-CEL (*p* < 0.01). In conclusion, DF extracted from linseed reduced apparent energy and fat digestibility and resulted in restriction of body weight gain in growing rats.

## 1. Introduction

Dietary fibers (DF) have received increasing attention for their potential role in weight regulation because high intakes of DF have been associated with a smaller weight gain in prospective observational studies [1,2,3]. Viscous soluble DFs in particular seem to play a role in short-term appetite regulation and food intake [4], whereas the long term effects of DF on body weight are less well documented. A meta-analysis of the effect of guar gum, a viscous DF, on body weight, concluded that no pronounced weight loss effect was experienced [5], whereas more consistent results were found for chitosan [6]. Chitosan interacts with intestinal fat digestion and absorption, thereby increasing faecal fat excretion, which is thought to be the main mechanism of action [6]. This attribute is also ascribed to viscous DF [7]. However, a recent study found that viscous fermentable guar gum did not inhibit weight gain in high-fat fed mice, whereas insoluble oat DF did inhibit weight gain, possibly due to differences in digestibility and energy utilisation of products deriving from gut fermentation [8].

Linseeds contain approximately 30% DF of which one third is water soluble. The majority of the water soluble DF belongs to a group of heterogenic polysaccharides consisting of neutral arabinoxylans and highly acidic rhamnose-containing polysaccharides present on the outside of the seed coat (the mucilage), which form highly viscous solutions when mixed with water [9,10]. These polysaccharides are, thus, easily extractable using only water. Alzueta and coworkers [11] investigated the effect of whole and demucilaged linseeds on growth in broiler chickens, and found that the DF-rich mucilage was responsible for the decreased weight gain observed in chickens fed linseed. They were also able to correlate to decreased nutrient utilization to mucilage [12]. In line with this, we previously found that addition of whole linseed to rye breads (18 g/day) significantly reduced the digestibility of fat and energy in humans [13]. It was also found that a linseed DF extract incorporated into breads decreased not only hunger, but also postprandial triacylglycerol supporting that linseed DF decrease lipid absorption [14]. However, we also found that food matrix may affect the magnitude of effect, as linseed DF in drinks were more effective than when incorporated into breads in increasing faecal fat excretion [15].

In the present study we tested the hypothesis that a diet with linseed DF derived from the mucilage fraction may decrease apparent energy and fat digestibility, thereby affecting body weight gain in growing rats.

## 2. Experimental Section

### 2.1. Animals and Study Design

The Danish Animal Experiments Inspectorate, Ministry of Justice, approved the protocol. Sixty growing male Wistar rats (Taconic Europe, Ry, Denmark), weighing 200 ± 3 g initially, were randomly allocated to six different dietary treatments (*n* = 10) and fed the experimental diets for 21 days. The cages were kept in a single room with controlled temperature (25 °C), relative humidity (50%–60%), and 12 h light and dark cycles. Fresh water was available and un-pelleted feed was supplied *ad libitum* (25 g feed supplied/day). During the last seven days of the trial the rats were housed individually in metabolic cages made of Perspex, with stainless steel mesh floors that allowed for quantitative collections of urine and faeces. No measurements were made on the first two days, in order to allow the rats to become accustomed to the cages. Faeces were then collected for the subsequent 5 days (balance period). Food and water containers were weighed before and after the balance period. Faecal samples were collected daily during the balance period, frozen, and stored at −20 °C. The rats were weighed to the nearest g on days 0, 4, 7, 9, 13, 16, 21 and sacrificed on day 22, following anaesthesia in a vapour of CO_2_. The gastrointestinal tract (GIT) was removed and stomach, small intestine, caecum and colon were weighed in all the animals. Stomach, small intestine, caecum and colon were then weighed again after removal of their contents. Heart, liver and abdominal fat pads were also removed and weighed in 5 animals in each group. In this sub-group of rats, a carcass homogenate of the whole rats (including fur and claws, but without GIT, blood and organs) was autoclaved with water for 1 h at 100 °C, blended into a smooth paste, freeze dried and analyzed for dry matter (DM), ash, N and fat. Hereafter, total body fat and protein percentage was calculated.

### 2.2. Experimental Diets

All six diets were high fat. The macronutrient composition of all six diets was the same, with ~110 g protein/kg DM, but they differed in DF content and source (Table 1). The DF were extracted from linseed hulls (provided by Natunola Health Inc., Ontario, Canada) as described elsewhere [14]. DF content of the linseed DF extract was measured according to the AOAC (No. 985.29) method [16] and used to calculate the content of linseed DF in the 5-LDF and 10-LDF diets. A table value on DF content in linseeds (28 g/100 g) [17] was used for calculation of DF content in the 5-WL and 5-GL diets. Based on the nutrient composition (protein, fat, DM, ash, DF, minerals and vitamins) of each ingredient, the feed mixtures were formulated to meet or exceed NRC recommendations for growing and reproducing rats [18].

### 2.3. Feed Analyses of Major Nutritional Compounds

Content of DM and ash in the diets was determined according to an AOAC method [19]. Nitrogen was determined by thermal conductivity after complete combustion at 1300 °C in pure oxygen (LECO CNS-2000, Carbon, Nitrogen and Sulphur Analyser, St. Joseph, MI, USA), and protein was calculated (*N* × 6.25). Gross energy was determined by bomb calorimetry using a LECO Ac 300 automated calorimeter system 789-500 (ISO 983:1998) [20]. Total non-starch polysaccharides (NSP) and their constituent sugars were determined as alditol acetates by gas-liquid chromatography for neutral sugars and by colorimetric determination for uronic acids using a modification of the Uppsala procedures as described by Bach Knudsen [21]. Klason lignin was measured as the insoluble residue after hydrolysis with 2 M H_2_SO_4_ and the content of total DF calculated as total NSP plus Klason lignin. Dietary fat (hydrochloric acid-fat) was extracted according to the Bligh and Dyer method using diethyl ether [22].

**Table 1:** Nutritional composition of the six experimental diets.

Diet	C	CEL	5-WL	5-GL	5-LDF	10-LDF
Ingredient, % (calculated)						
Maize starch	63.7	58.7	52.7	52.7	48.9	39.0
Linseed oil	10.00	10.00	6.45	6.45	9.20	8.40
Animal fat (pig)	10.00	10.00	6.45	6.45	9.20	8.40
Casein + 1% methionine	11.80	11.80	7.00	7.00	9.70	7.65
Vitamin mix	0.81	0.81	0.81	0.81	0.81	0.81
Mineral mix	3.70	3.70	3.70	3.70	3.70	3.70
Linseed	0.00	0.00	17.9	17.9	0.00	0.00
Linseed mucilage	0.00	0.00	0.00	0.00	13.5	27.0
Cellulose	0.00	5.00	5.00	5.00	5.00	5.00
Chemical composition (measured)						
DM, g/kg	973	989	973	975	979	976
Ash, g/kg DM	30.6	33.0	34.7	36.7	39.0	50.9
Protein, g/kg DM	111	110	110	109	107	111
Fat, g/kg DM	209	205	208	205	212	202
Gross E, kcal/kg DM	5280	5088	5232	5184	5179	5139
NSP, g/kg DM	14	68	76	76	99	158
Lignin, g/kg DM	0	3	16	16	9	31
DF g/kg DM	14	71	92	92	108	189

C: Control; DF: Dietary fiber; DM: Dry matter; E: Energy; 5-CEL: 5% DF from cellulose; 5-WL: 5% DF from whole linseed; 5-GL: 5% DF from ground linseeds; 5-LDF: 5% DF from linseed DF extract; 10-LDF: 10% F from linseed DF extract.

### 2.4. Analyses of Faecal and Tissue Samples

Faecal DM was determined by freeze drying. Faecal gross energy, fat, ash, and total carcass fat and nitrogen, were analyzed using the methods described above for feed analyses.

### 2.5. Calculations and Statistical Analyses

Apparent digestibility of energy and fat was calculated on the basis of recorded quantitative feed intake and excretion in faeces. All statistical analyses and calculations were performed using the Statistical Analysis System software package, version 9.3 (SAS Institute Inc., Cary, NC, USA). If the distribution of a variable was skewed it was log-transformed prior to analysis. A repeated measures ANCOVA was applied to examine the effect of diet and time and their interaction on weight gain with initial body weight as covariate. An ANCOVA was used to examine the effect of diet on all other dependent variables, in which final body weight was included as covariate, as these outcomes were assessed at the end of the trial. All data are presented as mean ± SE unless otherwise stated and the statistical significance level is defined as *p* < 0.05. The relationship between the digestibility of energy and fat and DF was analyzed by linear regression or a second-degree polynomial.

## 3. Results

**Figure 1:**
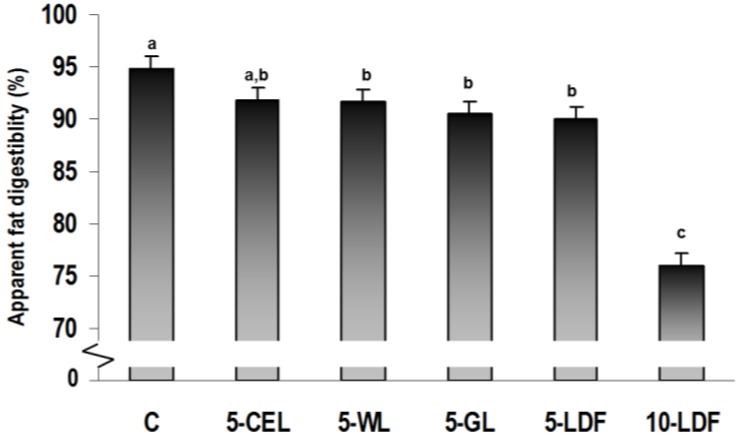
Apparent fat digestibility (% of intake) during the last 5 days of a 21 days feeding trial in rats fed six different diets (C = control; 5-CEL = 5% dietary fibers (DF) from cellulose; 5-WL = 5% DF from whole linseed; 5-GL = 5% DF from ground linseeds; 5-LDF = 5% DF from linseed DF extract; 10-LDF = 10% F from linseed DF extract) (Mean±SE) (*n* = 10). Mean values with different letters were significantly different (*p* < 0.05; ANCOVA, followed by least significant difference test).

Overall, there was an effect of diet on feed intake and apparent fat and energy digestibility (Table 2, Figure 1). Absolute feed intake differed between groups (*p* < 0.001), and was higher in the 5-CEL group compared to all diets expect 10-LDF, which also differed from the C diet. No other differences in feed intake were observed (Table 2). There was an effect of diet on fat (*p* = 0.013) and energy intake (*p* < 0.001), where the rats fed the 5-CEL diet had a higher intake compared to all other groups (*p* < 0.05). The largest effects were, however, observed on faecal volume (*p* < 0.001), where up to 8-fold increases could be observed in the 10-LDF group, which decreased with decreasing DF content: 10-LDF > 5-LDF> 5-GL = 5-WL > 5-CEL > C. Faecal energy excretion differed significantly between groups and a 6-fold increase was seen in rats fed the 10-LDF compared to rats fed the C diet (Table 2). Concomitantly, decreases in energy digestibility from 95.9% ± 0.5% in C to 76.1% ± 0.5% in 10-LDF fed rats were observed. Faecal fat excretion increased with increasing amounts of DF and the 10-LDF fed rats excreted 5-fold more fat compared to the C fed rats (*p* < 0.001), which reduced apparent fat digestibility from 94.9% ± 0.8% in the C group to only 74.3% ± 0.6% in the 10-LDF group (*p* < 0.001) (Figure 1).

**Table 2:** Nutrient digestibility, gastrointestinal tract (GIT) mass and content and body composition in rats fed diets with different source and content of DF (Mean ± SE, *n* = 10).

Outcome parameter	C		5-CEL		5-WL		5-GL		5-LDF		10-LDF *	
	Mean	SE	Mean	SE	Mean	SE	Mean	SE	Mean	SE	Mean	SE
Nutrient digestibility												
Feed intake (g/day)	14.9 ^c^	0.4	17.8 ^a^	0.3	15.7 ^bc^	0.3	16.1 ^bc^	0.3	15.4 ^bc^	0.4	16.5 ^ab^	0.4
Energy intake (kcal/day)	74.6 ^b^	1.9	90.4 ^a^	1.9	80.0 ^b^	1.9	81.5 ^b^	1.9	78.4 ^b^	1.9	82.6 ^b^	2.1
Fat intake (g/day)	3.10 ^b^	0.07	3.51 ^a^	0.07	3.24 ^b^	0.07	3.30 ^b^	0.07	3.27 ^b^	0.07	3.37 ^b^	0.08
Faecal volume (g/day)	0.92 ^e^	0.23	2.34 ^d^	0.23	3.29 ^cd^	0.24	3.33 ^c^	0.23	4.59 ^b^	0.23	7.71 ^a^	0.27
Energy excretion (kcal/day)	2.98 ^c^	0.55	7.85 ^b^	0.55	8.67 ^ab^	0.57	9.42 ^ab^	0.55	10.27 ^a^	0.56	19.08 ^a^	0.64
Fat excretion (mg/day)	154 ^c^	33	287 ^bc^	33	294 ^b^	33	349 ^b^	33	306 ^b^	33	829 ^a^	38
GIT mass and content												
Small intestine weight (g)	5.78 ^c^	0.24	5.11 ^c^	0.17	5.74 ^c^	0.24	6.23 ^b^	0.24	6.91 ^b^	0.23	9.11 ^a^	0.30
Small intestine content (g)	1.34 ^d^	0.25	2.66 ^c^	0.17	2.92 ^bc^	0.25	2.54 ^c^	0.25	3.66 ^b^	0.24	7.57 ^a^	0.31
Caecum weight (g)	0.80 ^b^	0.09	0.89 ^b^	0.07	0.78 ^b^	0.09	0.78 ^b^	0.09	1.17 ^b^	0.09	2.07 ^a^	0.12
Caecum content (g)	1.68 ^d^	0.46	2.79 ^cd^	0.32	3.45 ^cd^	0.46	3.69 ^bc^	0.46	5.47 ^b^	0.45	10.82 ^a^	0.57
Large intestine weight (g)	1.00 ^c^	0.09	1.08 ^c^	0.06	1.28 ^bc^	0.09	1.30 ^bc^	0.09	1.39 ^b^	0.08	1.76 ^a^	0.11
Large intestine content (g)	1.60 ^b^	0.33	1.73 ^b^	0.23	2.06 ^b^	0.33	2.21 ^b^	0.34	2.65 ^b^	0.33	4.88 ^a^	0.41
Body composition												
Final body weight (g)	282 ^a^	5.9	281 ^a^	5.9	285 ^a^	5.9	278 ^ab^	5.9	279 ^ab^	5.9	258 ^b^	6.2
Empty body weight (g)	275 ^a^	5.9	266 ^ab^	5.9	269 ^ab^	5.9	270 ^ab^	5.9	253 ^b^	5.9	222 ^c^	6.6
Abdominal fat (g) ^†^	14.1 ^b^	1.3	22.8 ^a^	0.9	14.5 ^b^	1.3	14.0 ^b^	1.3	11.9 ^b^	1.2	10.9 ^b^	1.6
Protein (%) ^†‡^	21.3	0.33	20.8	0.66	21.4	0.33	21.5	0.28	21.8	0.31	20.7	0.66
Fat (%) ^†‡^	13.1 ^ab^	0.99	16.5 ^a^	1.98	12.2 ^ab^	0.97	12.6 ^ab^	1.0	11.7 ^ab^	0.82	10.4 ^b^	0.82
Heart (mg) ^†^	949	29	1028	20	984	29	992	29	974	29	906	36
Liver (g) ^†^	10.56 ^a^	0.17	9.34 ^c^	0.11	10.11 ^ab^	0.16	9.99 ^ab^	0.17	9.58 ^bc^	0.15	9.73 ^bc^	0.20

GIT: gastrointestinal tract; DF: dietary fiber; C: Control; 5-CEL: 5% DF from cellulose; 5-WL: 5% DF from whole linseed; 5-GL: 5% DF from ground linseeds; 5-LDF: 5% DF from linseed DF extract; 10-LDF: 10% DF from linseed DF extract. ^a, b, c, d, e^ Mean values with different superscript letters within a row were significantly different (*p* < 0.05; ANCOVA, followed by least significant difference test). * *n* = 9 for nutrient digestibility data due to problems with sample collection; ^†^ *n* = 5, ^‡^ percentage of carcass weight.

The intermediate diets had a less pronounced effect on energy and fat digestibility, ranging from 86% to 96%. The digestibility of energy and fat in relation to DF was therefore better described by a second-degree polynomial than by a linear regression, and can be described as follows:

Apparent digestibility of energy = 96.95 − 0.0674*x* − 0.0002*x*^2^, *r*^2^ = 0.998 (1)

Apparent digestibility of fat = 94.43 + 0.0218*x* − 0.0007*x*^2^, *r*^2^ = 0.978 (2)

where *x* denotes DF.

There seemed to be an effect of linseed DF source on the digestibility of fat, with a decreased apparent digestibility with increased accessibility of DF (5-WL *vs.* 5-GL) and increased proportion of viscous DF (5-GL *vs.* 5-LDF) (Figure 1).

**Figure 2:**
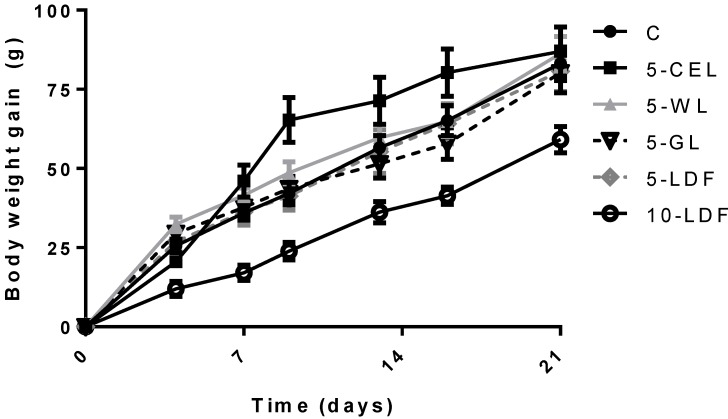
Weight gain during the 3 weeks feeding trial in rats fed six different diets (C = control; 5-CEL = 5% DF from cellulose; 5-WL = 5% DF from whole linseed; 5-GL = 5% DF from ground linseeds; 5-LDF = 5% DF from linseed DF extract; 10-LDF= 10% F from linseed DF extract) (Mean±SE) (*n* = 10). Significant overall effect of diet (*p* = 0.001) and time (*p* = 0.001) (repeated measures ANCOVA). Weight gain was significantly smaller in 10-LDF group compared to all other groups (*p* = 0.02; repeated measures ANCOVA followed by least significance difference).

An overall effect of diet (*p* = 0.05) and time (*p* < 0.001) on body weight was observed, but no interaction between time and diet was seen. However, only rats fed the 10-LDF had a significantly lower body weight after 3 weeks compared to diet C (−8.6%), 5-CEL (−8.3%), and 5-WF (−9.5%) (Figure 2, Table 2). No differences in weight gain of rats fed diets C, 5-CEL, 5-WF, 5-GF and 5-LDF were observed. Differences in weight were more pronounced for empty body weight. Rats fed 10-LDF had a lower empty body weight compared to rats in all other groups (*p* < 0.01) Rats fed 5-LDF differed from both C and 5-CEL fed rats (*p* < 0.05).

Weight of abdominal fat pads was highest in rats fed the 5-CEL diet. Total % fat of carcass weight was lower in the rats fed the 10-LDF diet compared to the 5-CEL fed rats (*p* < 0.05), whereas no differences were seen in % protein of carcass weight (*p* = 0.21). This means that the protein/fat ratio was almost doubled in the 10-LDF group compared to the 5-CEL group. Furthermore, the different DF sources and amounts significantly affected GIT weight (Table 2). GIT weight (without content) increased with increasing amounts of DF, and rats fed the 10-LDF diet had a larger total GIT than rats on all other diets (*p* < 0.001), probably reflecting elongation of the small intestine, caecum, and large intestine. Contents of the caecum, small and large intestine also increased, most so in the rats fed 5-LDF and 10-LDF diets (*p* < 0.001). As for a marker of apparent fat digestibility, both the amount of fat found in the small and large intestine tended to increase when the linseed were ground (5-WL *vs.* 5-GL), or when proportion of viscous DF was increased (5-GL *vs.* 5-LDF).

## 4. Discussion

Our hypothesis was that addition of linseed DF to animal feeds would suppress weight gain as a consequence of decreased apparent nutrient digestibility. Rats fed the 10-LDF diet had a lower daily weight gain compared to the rats fed the C, 5-CEL and 5-WL diets (*p* < 0.05), and empty body weight was lower compared to all other rats (*p* < 0.01). Apparent fat and energy digestibility were decreased by 10%–20% with the addition of a high dose of linseed DF to the feed, which could be modeled as a second-degree polynomial relationship. The second-degree relationship is undoubtedly due to the viscous nature of the linseed DF and its interference with the digestion and absorption of fat.

We have previously found linseed DF to be highly viscous in aqueous solution [15,23], but the viscosity of the gut lumen content of the rats was not assessed in the present study. However, our observations are in agreement with other studies, which have shown a reduction in weight gain with the addition of viscous DF to high fat diets [24,25]. Contrastingly, a recent study by Isken and colleagues (2010) suggested that soluble viscous DF was less effective for reducing body with than insoluble DF [8]. They found that guar gum resulted in a smaller faecal volume and excretion of energy compared to a diet with insoluble DF from oat hulls, probably due to the fermentation of the guar gum as reflected by increased H_2_-exhalation, higher energy digestibility and supposedly higher metabolisable energy content in the guar gum diet. We measured apparent energy digestibility, but not metabolisable energy. However, as weight gain was reduced and faecal volume and energy excretion significantly increased with the 10-LDF diet, it appears that viscosity played a larger role than fermentation of the linseed DF in the present study. Nonetheless the significantly larger contents of caecum of the 10-LDF fed rats suggest an increased bacterial fermentation. Our results confirm previous findings that addition of whole linseed to the feed reduces nutrient digestibility in broiler chickens [26] and ruminants [27]. Removal of the mucilage layer of the seeds offset the effect on apparent energy and fat digestibility in broiler chickens [11], clearly indicating that the DF component is responsible for the observed effects. Viscosity of the jejunal digesta of the chickens correlated with the digestibility of nutrients [28], thus the physiological effects appear to be closely linked to the viscous properties of the linseed DF. As in the present study, a non-linear increase in viscosity with increased concentration of DF was found [28].

The higher feed intake in the 5-CEL group compared to the other groups did not result in increased weight gain. The small differences in feed intake between 10-LDF and 5-LDF, 5-GL and 5-WL suggest that the rats may have tried to compensate for the lower nutrient digestibility resulting from the 10-LDF diet. However, this was not sufficient to achieve a growth rate in the 10-LDF group similar to that of the other groups. The high dose of DF in the 10-LDF group did not influence the protein digestibility to such a degree that it affected muscle mass; but the percentage of body fat decreased. Addition of linseed DF to the feed also caused a significant increase in weight of total GIT and different segments of the GIT. This may be due to (1) increased microvilli length and density; or (2) an elongation of the GIT as a means of compensation for decreased nutrient utilization, although neither length of the different segments or gut morphology were not recorded in the present study. Our findings are in accordance with Zhao *et al.* [29], who found an almost two-fold increase in weight of the GIT, with a high *vs.* low DF diet (257 g * vs.* 56 g DF/DM). DF may also affect the weight of the liver, kidney, and heart, as observed in other animal studies [29,30,31], but we did not see this here.

We hypothesized that similar doses of DF provided as whole or ground linseed, or as extract, would affect nutrient digestibility differently due to differences in botanical integrity, particle size, and DF type. In the 5-WL and 5-GL diets the DF were a mix of soluble and insoluble DF, whereas the 5-LDF diet contained only water-extracted soluble DF. These differences in DF type did not result in any significant differences in body weight gain, whereas apparent fat digestibility, faecal volume and weight of intestinal contents was larger when rats were fed extracted viscous linseed DF compared to a mix of DF types. That fat digestibility was affected to some degree indicates that luminal viscosity may have differed, although this was not verified in the current study. The differences in luminal content, particularly in the caecum and large intestine, suggest that fermentation was increased with a higher viscous DF content, as increased luminal content is believed to result from increased bacterial mass [32]. Contrastingly, there were no detectable differences in luminal content between whole and ground linseeds. We speculate that results might have been different if the study had been conducted in humans, as whole linseed probably pass through the human GIT, at least to some extent, in their intact structure. This is not the case in rats, where the seeds are chewed.

The digestive tract varies greatly between species, but previous studies have shown that the utilization of energy, protein, fat, and NSP, are reasonably comparable in rats and humans [33,34,35], although the ability to ferment cellulose differs between rats and humans. It is therefore not appropriate to extrapolate our findings of reduced nutrient digestibility to humans. It is worth noting that the daily dose of DF fed to the rats in the present study exceeds that of a normal DF diet in humans, as a diet containing 10%–15% NSP of DM would correspond to a daily intake of 40–60 g of DF in humans. Furthermore, this study was performed in growing rats, where a high proportion of absorbed nutrients are used for growth. The results obtained on body weight gain should be interpreted with great caution in terms of potential effects on body weight in humans, and the fact that the impact of DF on appetite regulation may differ between rats and humans should also be taken into account.

## 5. Conclusions

In conclusion, a high dose of DF extracted from linseed increased faecal fat and fat excretion, and resulted in decreased body weight gain in growing rats, whereas diets with similar DF content for the whole or ground linseed or DF extracted from linseed did not affect body weight differently, despite differences in apparent fat and energy digestibility. The present findings should be regarded as a proof of concept for this new source of extracted DF. We believe that linseed DF may be a useful food ingredient for its effect on energy balance, also in humans, although this needs to be confirmed in long-term human intervention trials.

## References

[1] Bazzano L.A., Song Y., Bubes V., Good C.K., Manson J.E., Liu S. (2005). Dietary intake of whole and refined grain breakfast cereals and weight gain in men. Obes. Res..

[2] Koh-Banerjee P., Franz M., Sampson L., Liu S., Jacobs D.R., Spiegelman D., Willett W., Rimm E. (2004). Changes in whole-grain, bran, and cereal fiber consumption in relation to 8-y weight gain among men. Am. J. Clin. Nutr..

[3] Liu S., Willett W.C., Manson J.E., Hu F.B., Rosner B., Colditz G. (2003). Relation between changes in intakes of dietary fiber and grain products and changes in weight and development of obesity among middle-aged women. Am. J. Clin. Nutr..

[4] Wanders A.J., van den Borne J.J., de Graaf C., Hulshof T., Jonathan M.C., Kristensen M., Mars M., Schols H.A., Feskens E.J. (2011). Effects of dietary fibre on subjective appetite, energy intake and body weight: A systematic review of randomized controlled trials. Obes. Rev..

[5] Pittler M.H., Ernst E. (2001). Guar gum for body weight reduction: Meta-analysis of randomized trials. Am. J. Med..

[6] Jull A.B., Ni M.C., Bennett D.A., Dunshea-Mooij C.A., Rodgers A. (2008). Chitosan for overweight or obesity. Cochrane Database. Syst. Rev..

[7] Pasquier B., Armand M., Castelain C., Guillon F., Borel P., Lafont H., Lairon D. (1996). Emulsification and lipolysis of triacylglycerols are altered by viscous soluble dietary fibres in acidic gastric medium *in vitro*. Biochem. J..

[8] Isken F., Klaus S., Osterhoff M., Pfeiffer A.F.H., Weickert M.O. (2010). Effects of long-term soluble *vs.* insoluble dietary fiber intake on high-fat diet-induced obesity in C57BL/6J mice. J. Nutr. Biochem..

[9] Warrand J., Michaud P., Picton L., Muller G., Courtois B., Ralainirina R., Courtois J. (2005). Structural investigations of the neutral polysaccharide of *Linum usitatissimum* L. seeds mucilage. Int. J. Biol. Macromol..

[10] Cui W., Mazza G., Biliaderis C.G. (1994). Chemical structure, molecular size distributions, and rheological properties of flaxseed gum. J. Agric. Food Chem..

[11] Alzueta C., Rodriguez M.L., Cutuli M.T., Rebole A., Ortiz L.T., Centeno C., Trevino J. (2003). Effect of whole and demucilaged linseed in broiler chicken diets on digesta viscosity, nutrient utilisation and intestinal microflora. Br. Poult. Sci..

[12] Berggren A.M., Björck I.M.E., Nyman E.M.G.L., Eggum B.O. (1993). Short-chain fatty acid content and pH in caecum of rats given various sources of carbohydrates. J. Sci. Food Agric..

[13] Kristensen M., Damgaard T.M., Sorensen A.D., Raben A., Lindelov T.S., Thomsen A.D., Bjergegaard C., Sorensen H., Astrup A., Tetens I. (2008). Whole flaxseeds but not sunflower seeds in rye bread reduce apparent digestibility of fat in healthy volunteers. Eur. J. Clin. Nutr..

[14] Kristensen M., Savorani F., Christensen S., Engelsen S.B., Bügel S., Toubro S., Tetens I., Astrup A. (2011). Flaxseed dietary fibers suppress postprandial lipemia and appetite sensation in young men. Nutr. Metab. Cardiovasc. Dis..

[15] Kristensen M., Jensen M.G., Aarestrup J., Petersen K.E., Sondergaard L., Mikkelsen M.S., Astrup A. (2012). Flaxseed dietary fibers lower cholesterol and increase fecal fat excretion, but magnitude of effect depend on food type. Nutr. Metab. (Lond.).

[16] Prosky L., Asp N.G., Furda I., DeVries J.W., Schweizer T.F., Harland B.F. (1985). Determination of total dietary fiber in foods and food products: Collaborative study. J. Assoc. Off. Anal. Chem..

[17] Saxholt E., Christensen A.T., Møller A., Hartkopp H.B., Hess Y., Hels O. (2008). Danish Food Composition Databank, Revision 7.

[18] National Research Council (1995). Nutrient Requirements of Laboratory Animals.

[19] AOAC (1990). Official Methods of Analysis.

[20] Hansen B. (1989). Determination of nitrogen as elementary N, an alternative to Kjeldahl. Acta Agric. Scand..

[21] Bach Knudsen K.E. (2009). Carbohydrate and lignin contents of plant materials used in animal feeding. Anim. Feed Sci. Technol..

[22] Lauridsen C., Christensen T.B., Halekoh U., Jensen S.K. (2007). Alternative fat sources to animal fat for pigs. Lipid Technol..

[23] Ibrugger S., Kristensen M., Mikkelsen M.S., Astrup A. (2012). Flaxseed dietary fiber supplements for suppression of appetite and food intake. Appetite.

[24] Artiss J.D., Brogan K., Brucal M., Moghaddam M., Jen K.-L.C. (2006). The effects of a new soluble dietary fiber on weight gain and selected blood parameters in rats. Metabolism.

[25] Woo M.N., Bok S.H., Lee M.K., Kim H.J., Jeon S.M., Do G.M., Shin S.K., Ha T.Y., Choi M.S. (2008). Anti-obesity and hypolipidemic effects of a proprietary herb and fiber combination (S&S PWH) in rats fed high-fat diets. J. Med. Food.

[26] Gonzalez-Esquerra R., Leeson S. (2000). Studies on the metabolizable energy content of ground full-fat flaxseed fed in mash, pellet, and crumbled diets assayed with birds of different ages. Poult. Sci..

[27] Petit H.V. (2002). Digestion, milk production, milk composition, and blood composition of dairy cows fed whole flaxseed. J. Dairy Sci..

[28] Dikeman C.L., Murphy M.R., Fahey G.C. (2006). Dietary fibers affect viscosity of solutions and simulated human gastric and small intestinal digesta. J. Nutr..

[29] Zhao X., Jorgensen H., Eggum B.O. (1995). The influence of dietary fibre on body composition, visceral organ weight, digestibility and energy balance in rats housed in different thermal environments. Br. J. Nutr..

[30] Sklan D., Smirnov A., Plavnik I. (2003). The effect of dietary fibre on the small intestines and apparent digestion in the turkey. Br. Poult. Sci..

[31] Dvir I., Chayoth R., Sod-Moriah U., Shany S., Nyska A., Stark A.H., Madar Z., Arad S.M. (2000). Soluble polysaccharide and biomass of red microalga *Porphyridium* sp. alter intestinal morphology and reduce serum cholesterol in rats. Br. J. Nutr..

[32] Jensen B.B., Jorgensen H. (1994). Effect of dietary fiber on microbial activity and microbial gas production in various regions of the gastrointestinal tract of pigs. Appl. Environ. Microbiol..

[33] Bach Knudsen K.E., Wisker E., Daniel M., Feldheim W., Eggum B.O. (1994). Digestibility of energy, protein, fat and non-starch polysaccharides in mixed diets: Comparative studies between man and the rat. Br. J. Nutr..

[34] Sembries S., Dongowski G., Mehrlander K., Will F., Dietrich H. (2004). Dietary fiber-rich colloids from apple pomace extraction juices do not affect food intake and blood serum lipid levels, but enhance fecal excretion of steroids in rats. J. Nutr. Biochem..

[35] Wisker E., Bach Knudsen K.E., Daniel M., Feldheim W., Eggum B.O. (1996). Digestibilities of energy, protein, fat and nonstarch polysaccharides in a low fiber diet and diets containing coarse or fine whole meal rye are comparable in rats and humans. J. Nutr..

